# Microwave-Treated Physically Cross-Linked Sodium Alginate and Sodium Carboxymethyl Cellulose Blend Polymer Film for Open Incision Wound Healing in Diabetic Animals—A Novel Perspective for Skin Tissue Regeneration Application

**DOI:** 10.3390/pharmaceutics15020418

**Published:** 2023-01-27

**Authors:** Saima Mahmood, Nauman Rahim Khan, Ghulam Razaque, Shefaat Ullah Shah, Memuna Ghafoor Shahid, Hassan A. Albarqi, Abdulsalam A. Alqahtani, Ali Alasiri, Hafiz Muhammad Basit

**Affiliations:** 1 Gomal Centre for Pharmaceutical Sciences, Faculty of Pharmacy, Gomal University, DIKhan 29050, Khyber Pakhtunkhwa, Pakistan; 2 Department of Pharmacy, Kohat University of Science and Technology, Kohat 26000, Khyber Pakhtunkhwa, Pakistan; 3 Faculty of Pharmacy, University of Baluchistan, Quetta 87300, Baluchistan, Pakistan; 4 Department of Botany, Government College University, Lahore 54000, Punjab, Pakistan; 5 Department of Pharmaceutics, College of Pharmacy, Najran University, Najran 55461, Saudi Arabia; 6 Akhtar Saeed College of Pharmacy, Bahria Golf City, Rawalpindi 46220, Punjab, Pakistan

**Keywords:** polymeric film, diabetes mellitus, skin regeneration, wound healing, sodium alginate, sodium carboxy methyl cellulose, microwave, cross-linking

## Abstract

This study aimed at developing the microwave-treated, physically cross-linked polymer blend film, optimizing the microwave treatment time, and testing for physicochemical attributes and wound healing potential in diabetic animals. Microwave-treated and untreated films were prepared by the solution casting method and characterized for various attributes required by a wound healing platform. The optimized formulation was tested for skin regeneration potential in the diabetes-induced open-incision animal model. The results indicated that the optimized polymer film formulation (MB-3) has significantly enhanced physicochemical properties such as high moisture adsorption (154.6 ± 4.23%), decreased the water vapor transmission rate (*WVTR*) value of (53.0 ± 2.8 g/m^2^/h) and water vapor permeability (*WVP*) value (1.74 ± 0.08 g mm/h/m^2^), delayed erosion (18.69 ± 4.74%), high water uptake, smooth and homogenous surface morphology, higher tensile strength (56.84 ± 1.19 MPa), and increased glass transition temperature and enthalpy (through polymer hydrophilic functional groups depicting efficient cross-linking). The in vivo data on day 16 of post-wounding indicated that the wound healing occurred faster with significantly increased percent re-epithelialization and enhanced collagen deposition with optimized MB-3 film application compared with the untreated group. The study concluded that the microwave-treated polymer blend films have sufficiently enhanced physical properties, making them an effective candidate for ameliorating the diabetic wound healing process and hastening skin tissue regeneration.

## 1. Introduction

Skin tissue regeneration following wounds (trauma, severe burns, venous stasis, decubitus/pressure, and diabetic ulcers) has been a challenging task for biomedical scientists, especially the aesthetic outcome [1,2], and a great deal of attention has been concentrated on the research and development of polymer films as wound dressings and drug delivery systems [3,4]. The diabetic wound has been a primary health concern where the normal wound healing process is delayed due to a persistently high blood glucose level, high oxidative stress, and a compromised immune system [5], which makes it imperative to develop scaffolds with properties inculcated that are required by ideal wound dressing materials [1,6].

Wound healing is a cascade of related molecular events that work jointly to reinstate cellular function and tissue integrity [7]. These events efficiently take place in normal, healthy human beings. Still, in certain conditions, such as poor nourishment or diseases such as diabetes, these events are impeded, resulting in chronic or hard-to-heal wounds [8], which are often complicated by secondary bacterial infection, thus not only prolonging the treatment period but also increasing the treatment cost to the patient. Hence adequate blood supply to the wound area, hygiene, the absence of necrotic scraps, sufficient moisture balance, avoidance of microbial infection, and good exudate management are key factors to ensure speedy recovery of the damaged skin tissue [9].

Polymeric films have long been used as a cost-effective strategy to hasten skin tissue regeneration following damage. Hence, various polymers have been used for the purpose, such as cellulose [10], sodium carboxymethyl cellulose [11,12], chitosan [13,14], sodium alginate [3,15,16], gelatin [17], collagen [18], agar [19], pectin [20], dextran [21], carrageenan [22], and hyaluronic acid [23]. The lone use of polymer for film dressing has always been devoid of properties required of an ideal wound healing platform [24], such as efficient moisture adsorption, prevention of trans-epidermal water loss, efficient gaseous exchange between the wound bed and external environment with controlled pore size preventing bacterial infiltration [25,26], enhanced mechanical properties, and delayed degradation [24]. These demerits can be overcome by cross-linking the polymeric films, which not only results in a mechanically strong film but also enables modification of one or more physicochemical properties of the film such as tensile strength, strain, higher temperature performance, cell-matrix interactions, gas permeation reduction, shape memory retention, and resistance to enzymatic and chemical degradation [27]. 

Crosslinking is described as the generation of physical or chemical linkage between polymer chains, which is generally an easy way to amend the biological, degradation, and mechanical properties of the polymer [28]. This is regarded to happen through functional groups by the generation of additional linkages through either chemical (covalent) or physical bond formation (hydrogen bonds, electrostatic interactions, and/or Vander Waal’s forces, etc.) [29]. The majority of materials that undergo no treatment do not hold the required degradation and mechanical properties of the engineered platform; consequently, there is a severe demand to augment those properties by use of smaller molecules named “crosslinking agents” or “crosslinkers” [24]. The prime objective of crosslinking is to enhance the biomechanical properties of the scaffold by creating a compact network in a polymer matrix [30,31,32]. Ionic cross-linking has been mainly used for this purpose [33,34] by using compounds such as glutaraldehyde, formaldehyde, epoxy compounds, and dialdehyde. It is regarded as a highly versatile method [31]. However, chemical cross-linking is an extremely flexible technique to improve the mechanical properties of polymers, thus offering improved mechanical stability compared with physically cross-linked polymers. Still, these cross-linking compounds are frequently toxic, exhibit undesirable effects, may exert toxic reactions that result in cytotoxicity, can induce unwanted reactions with the scaffold surface, and are not environmentally responsive [24,35,36]. Thus, their complete removal from the reaction mixture makes it prone to induce necrosis in the wound [37,38]. 

To address the demerits associated with the use of chemical cross-linkers, physical methods of crosslinking were introduced, such as ultraviolet irradiation [39], electron beam irradiation [31], exposing the polymers to X-rays [40], alpha-rays [41], gamma-rays [42,43], and microwaves [44,45]. In physical cross-linking, polymers may be efficiently cross-linked without using any exogenous crosslinking agent, thus reducing the hazard of chemical adulteration or chemically raised harmfulness [35]. Due to the absence of chemical crosslinking agents, biomedical safety is the primary benefit of a physical crosslink, evading possible cytotoxicity from unreacted chemical crosslinkers [46]. Physical crosslinking is through physical interactions such as crystallization, protein interaction, hydrogen bonding, hydrophilic/hydrophobic interaction, stereocomplex formation/complexation, and ionic interactions, which help keep the polymer chains together [36,47].

Microwaves are electromagnetic waves characterized by frequency in the range of 300 MHz to 300 GHz [38] and have long been used for polymerization reactions [48], for the synthesis of an extensive range of polymers [49], for the development of hydrogel scaffolds [50], for the fabrication of hydrogel [51] and nanoparticle gel [52], for improving the mechanical properties of the polymer [53], for polymer modification and composite film formulation [54,55], and for polymeric films [56,57]. Microwave interacts with polar functional groups in a volumetric manner, thereby initiating polymer cross-linking through their polar moieties, with the added merits of excluding the use of catalysts or additives to start the reaction and simplicity of irradiation methods; the crosslinking point may be controlled effortlessly by differing the dose of irradiation [36]. Multiple studies have been performed to evaluate the role of the microwave as a crosslinker which significantly enhanced the physicochemical attributes of the resultant product such as titanium dioxide nanoparticles containing cross-linked chitosan hydrogel scaffold [50], microwave-aided bioactive chitosan scaffold containing gold nanoparticles [58], poly acrylic acid, polyvinyl alcohol, polyacrylamide, hydroxyl ethyl cellulose and polymethyl vinyl-ether-alt-maleic anhydride cross-linked hydrogels without the use of monomers [51], chitosan/polyvinyl alcohol silver nanoparticles gel [52], poly-lactic acid and poly-glycolic acid blend [53], microcrystalline corn-straw cellulose cross-linked film [55], hydroxy propyl methyl cellulose and poly(vinylpyrrolidone) composite films [45], and polyvinyl alcohol and tartaric acid films [56].

Sodium alginate (SA) is a water-solvable hydrocolloid that is obtained from brown seaweed and is composed of (1–4)-linked -d-mannuronic acid and -l-guluronic acid units [59]. The significant abilities of this polymer, such as biocompatibility, non-toxicity, reproducibility, and biodegradation, have directed its usage in several fields, including pharmaceutical additives, tissue engineering materials, food, and biology or enzyme carriers [60]. It has also been investigated for its wound-healing potentials, such as the development of biocompatible povidone-iodine-containing sodium alginate film for enhancement of ulcer healing [16], sodium alginate and gelatin hydrogels as wound dressings [19], and the formation of PVA-sodium alginate hydrogel membrane containing bFGF-entrapped microspheres for enhanced wound healing [61]. 

Sodium Carboxymethyl cellulose (NaCMC), a biopolymer, is one of the derivatives of cellulose that is developed by substituting the hydroxyl group (-OH) with the carboxymethyl (-COOH2CH-) group, where both units are linked to each other by β 1 and 4 glycosidic linkages [62,63]. It possesses excellent swelling and water-absorbing properties [64] and is biologically inert and biocompatible [65,66]. It also finds widespread applications in reduction, flocculation, detergents, paper, textiles, food, and drug formulation [67]. It also has the added merit of being safe, non-toxic, and non-sensitizing, and hence also finds applications in food, cosmetic, pharmaceutical, and biomedical applications, as well as wound management [68,69]. It also possesses excellent film-forming properties [70] and has been widely studied for wound-healing applications [62,68,71,72,73].

Both sodium alginate and Na-CMC have been extensively studied from the perspective of wound healing [16,25,62,74,75,76], but their sole use has been associated with demerits such as high water vapor transmission [77,78], fast erosion due to high hydrophilicity [79], low absorbability [78,80], permeability to bacteria [81], low gaseous exchange between the wound bed and external environment [82,83], and poor mechanical strength [84,85,86,87], which necessitates the development of their blends [79]. 

This project aimed to develop physically cross-linked sodium alginate and NaCMC films through microwave treatment, analyze them for physicochemical attributes, and perform in vivo testing in diabetes-induced animal models for rapid healing following open incision wound infliction. Blended sodium alginate/NaCMC films have been explored as prospective combinations that can be physically crosslinked using the microwave. Their combination was optimized by varying the microwave treatment time while keeping the concentration of both polymers constant. The optimized combination was tested for its ability to regenerate skin tissue in diabetic animals following open incision wound infliction.

## 2. Materials and Methods

### 2.1. Materials

Polysorbate 80 (tween-80, purity ~99%), disodium hydrogen orthophosphate (purity ~99%), sodium chloride (purity ~99%), sodium carboxymethyl cellulose (Na-CMC, purity ~99%, molecular weight 262.19 g/mol, high viscosity:1500–3000 centipoise of 1% solution in water at 25 °C), and monobasic potassium phosphate (purity ~98%) were procured from Sigma-Aldrich (St. Louis, MO, USA), while hydrochloric acid (purity ~35%) was purchased from Merck, Darmstat, Germany. Sodium alginate (purity ~99%, molecular weight 216.12 g/mol, viscosity: 15–25 centipoise of 1% solution in water) was bought from Sinopharm Chemical Reagent Co., Ltd., Shanghai, China. Polyethylene glycol-400 (PEG-400, purity ~99%) and glycerol (purity ~99%) were kindly provided by Bio-Labs (Islamabad, Pakistan). All chemicals were used without any further processing or purification.

### 2.2. Methods

#### 2.2.1. Film Formulation

Bi-polymeric blended films composed of sodium alginate and Na-CMC were developed by solution casting technique as described earlier [33]. Briefly, sodium alginate and Na-CMC were separately dissolved in enough deionized water to prepare (2% *w*/*w*) solutions. Both solutions were then added with glycerol (2% *w*/*w*), tween 80 (0.1% *w*/*w*) and PEG-400 (0.05% *w*/*w*) and thoroughly mixed to ensure homogeneity. Both polymer solutions were mixed in a ratio of 60:40 (60 parts sodium alginate and 40 parts Na-CMC) and subjected to microwave treatment for different time intervals (1 and 3 min) at fixed power of 500 watts and a fixed frequency of 2450 MHz, utilizing a commercially available microwave oven (LG, MS2022D, Beijing, China). Following microwave treatment, a total of 50 g of bubble-free polymer mixture was transferred into petri dishes (Ø 34.30 mm) and dried in a convection oven (SH SCIENTIFIC, Model: SH- DO-100NG, Sejong, Korea) at 40 °C for 72 h or until complete dryness. 

The dried polymeric films were detached from petri dishes and kept in a desiccator until they were subjected to several physicochemical characterization tests. The untreated blend films were developed similarly for comparison. The formulation ingredients and microwave treatment conditions are depicted in Table 1.

#### 2.2.2. Moisture Adsorption

An already-reported method [88] was used to determine moisture adsorption. Concisely, the film was sliced into 2.5 × 3 cm pieces and accurately weighed. The film pieces were dehydrated in a desiccator with anhydrous calcium sulfate (CaSO_4_) at virtual relative humidity (RH) of 0% for 48 h. Following complete desiccation, the dried film pieces were weighed again and incubated again with a saturated solution of potassium sulfate in a desiccator at 25 ± 2 °C with relative humidity maintained at 97 ± 2% for an additional 48 h to allow the films to become fully hydrated and were weighed again. The percent (%) moisture adsorption was determined using the following equation.

*MA* (%) = (*Wt* − *Wi*)/*Wi* × 100 (1)

*Wt* = Final weight after rehydration, and *Wi* = Initial weight after dehydration.

The test was repeated three times and results were averaged with ± standard deviation.

#### 2.2.3. Water Vapor Transmission Rate (*WVTR*) and Water Vapor Permeability (*WVP*)

The water vapor transmission rate (*WVTR*) through the polymeric film was assessed by the ASTM method with some modifications using a plastic bottle with its mouth area determined [89]. The film was cut in size according to the mouth of the bottle. The bottle was filled with a 30 mL saturated potassium chloride solution, and the film was tied onto its mouth with an adhesive. The whole system was initially weighed and then placed in a desiccator containing calcium chloride at room temperature with RH maintained at 1.5%. The system’s weight was determined hourly for up to 8 h. The loss in weight is considered equal to the amount of water transmitted across the film, which was absorbed by desiccant material (CaCl_2_). The thickness of the films was determined using a micrometer screw gauge. The values were placed in equations to determine the WVT, which was estimated by dividing the slope of a linear regression of weight loss vs. time by *film area*, and the *WVP* (gm/m^2^ s Pa) was determined by using an equation.

(2) $$WVTR=\frac{Slope}{Film\;area}$$

The *slope* is the slope of the graph calculated from the weight loss vs. time curve, and the *film area* was 0.000903 m^2^.

(3) $$WVP=\frac{WVTR\times T}{\Delta P}$$

*T* is the mean film thickness (mm) and Δ*P* is the partial water vapor pressure difference (mmHg) through two sides of the film sample (the partial vapor pressure of water at 25 °C = 23.73 mmHg).

The experiment was repeated three times, and results averaged with ± SD.

#### 2.2.4. Erosion and Water Uptake

Briefly, film pieces were cut, each having a 3 × 2.5 cm dimension. Then dry film pieces were weighed and placed in 20 mL PBS of pH 7.4 in a petri dish. After that, it was incubated at 37 ± 3 °C in a convection oven (SH SCIENTIFIC, Model: SH- DO-100NG, Sejong-si, Republic of Korea). The film pieces were taken from the petri dish at a specific time interval (every 5 min), blotted dry, and weighed again. The same was repeated for the entire incubation time of over 20 min. After 20 min, the buffer solution was discarded, and the same film pieces were dried in an oven at 40 ± 2 °C for 5 days. After 5 days, the oven-dried weight was taken, and the following relations were used to calculate percent erosion (*E*%) and percent water uptake (*WU*%). The test was executed in triplicate, and the results were averaged with standard deviation.

(4) $$E\;\left(\%\right)=\frac{Wi-Wt\left(d\right)}{Wi}\times 100$$

(5) $$WU\;\left(\%\right)=\frac{Wt-Wt\left(d\right)}{Wt\left(d\right)}\times 100$$

*Wi* = weight of film before immersion, *Wt* (*d*) = dry weight of film taken at time *t*, and *Wt* = wet weight of film at time *t*.

#### 2.2.5. Morphology

Scanning electron microscopy was used to analyze the film’s surface morphology using an ultra-high-resolution field-emission scanning electron microscope (UHR-FESEM, MERLIN/344999-9001-030, Zeiss, Aalen, Germany). Each film was sliced into a 3 × 3 mm piece, which was then attached to a stub via double-sided adhesive carbon tape. The samples were then placed in the microscope chamber after being subjected to a 5-min gold sputter coating procedure (QUORUM Sputter Coater Q150R S, Quorum, Lewes, UK), followed by SEM examination at a 10-KV accelerating voltage. Using the smartTiff tool, the photographs of corresponding parts were taken at magnification powers of 100, 500, 1000, 2000, and 3000× [34].

#### 2.2.6. Tensile Strength

Using a universal testing device (Testometrics, Rochdale, UK), the polymeric films’ ultimate tensile strength was assessed at 25 ± 1 °C. The polymeric film’s tensile strength was measured employing a texture analyzer after being trimmed into rectangular-shaped strips. For each film sample, three rectangular-shaped strips of 7.5 cm in length and 3.5 cm in width were trimmed, and they were then fastened between the machine’s grips. The initial grip distance was set to 50 mm, and the crosshead speed was set to 5 mm/min. The sample was pulled with a 50 N load [34]. The most significant breaking force was noted. The results were averaged across three replicated tests.

#### 2.2.7. Differential Scanning Calorimetry (DSC)

The films’ thermal characteristics were assessed using differential scanning calorimetry (DSC; PerkinElmer Thermal Analysis, USA) [89]. A 5–7 mg film sample was taken in the sample pan. The reference pan was left empty. Melting transition temperatures (T) of various films were recorded while continuously purging with N_2_ gas with a 40 mL/min flow rate and a heating scan rate of 10 °C/min from 0–400 °C. Each film peak’s transition temperature and enthalpy (∆H) values were calculated in triplicate, and the results were averaged.

#### 2.2.8. Vibrational Spectroscopic Analysis

An ATR-FTIR spectrophotometer (UATR TWO, Perkin Elmer, Beaconsfield, UK) [89] captured the dried polymeric films’ distinctive peaks. Each film was laid on the diamond crystal’s surface and secured to guarantee close contact and great sensitivity. All samples were scanned with a 2 min acquisition time spanning the 400 to 4000 cm^−1^ wavenumber range. Results were averaged after three analyses of each sample.

#### 2.2.9. In Vivo Wound Healing

Healthy male Sprague–Dawley rats with a weight range of 200–250 g were procured and acclimatized/adjusted for 14 days with an easy approach to water and food at a temperature of 19 to 23 °C with a 12-h dark-light cycle. Before the diabetes induction, the rats abstained from eating for 24 h with free access to water. They were weighed, and their fasting blood sugar levels were determined using a glucometer (CodeFree, SD Biosensor, Korea). A single dose of freshly prepared streptozotocin solution was intraperitoneally injected in rats at a dosage of 50 mg/kg body weight of the animal. The blood sugar levels of the animals were monitored starting on day 3 of the streptozotocin injection [90], and rats were considered people with diabetes when their blood sugar levels were >250 mg/dL [91]. Following the diabetes induction, the diabetic rats were divided randomly into two groups (*n* = 8 for each group), i.e., untreated and polymeric film groups. The rats were anesthetized by I/P injection of a mixture of xylazine (10 mg/kg) and ketamine (100 mg/kg), and the back hair of the rats was shaved. An open incision wound was inflicted on a mid-dorsal thoracic section of the rats with the help of sterilized forceps and surgical scissors. Following the infliction of the wound, the treatments were applied to the injured part, covered with sterile gauze, and adhered with 3M adhesive tape. The untreated group received only the gauze application, while the polymeric film group received only the (3 × 3 dimensional) film piece. The treatments were applied daily until complete wound healing was observed. The institutional Ethical Review Board approved the animal study protocol, vide reference number: 502/QEC/GU, dated: 29 March 2019, Gomal University Pakistan.

The photographs of wounds were taken by a Canon D5200 camera (Tokyo, Japan) on days 0, 3, 7, 14, and 16 post-administration to record the surface morphology of the wound. The wound size was investigated by Image J software (version 1.53K, US National Institutes of Health, Bethesda, MD, USA). The % re-epithelization was then estimated using the following relation.

(6) $$Re-epithelialization\;\left(\%\right)=\frac{Wound\;size\;at\;time\;0-Wound\;size\;at\;time\;t}{Wound\;size\;at\;time\;0}\times 100$$

#### 2.2.10. Physicochemical Characterization of Skin Samples

##### Thermal Analysis

To estimate changes produced in the lipid and protein regions of skin with film treatment compared with the control group, the skin samples with wounds were also exposed to thermal analysis employing DSC (Perkin Elmer, Thermal Analysis, Boston, MA, USA). In a nutshell, a precisely measured 3 mg of full-thickness skin-containing wound was trimmed/cut with great care and enclosed in a standard aluminum pan before being subjected to thermal analysis at temperatures ranging between 30–180 °C at a heating rate of 10 °C/min, under constant pulses of nitrogen gas at a 40 mL/min flow rate. For the lipidic and protein regions, the melting temperature and enthalpy were noted. Results were averaged after at least three separate analyses of each sample.

##### Tensile Strength

The tensile strength of the excised skin samples was measured after they were cut into strips of 5 cm in length and 2.5 cm in width (Testometric M-500, Rochdale, UK). The strips were fastened between the lower and upper jaws of the tensiometer and perpendicularly pulled/strained with loads of 30 kg at test speeds of 5 mm/s and 10 mm/s, respectively. The greatest power necessary to rupture the skin sample and the breaking point were noted. Test of every skin specimen was performed thrice, and results were averaged.

##### Vibrational Spectroscopy

ATR-FTIR (UATR TWO, Perkin Elmer, Buckinghamshire, UK) was used to record the vibrational spectra of the dermal layer of skin samples from treated and untreated animal groups with a resolution of 16 cm^−1^ and acquisition/exposure duration of 2 min. The corresponding ATR-FTIR spectra were compared to determine the degree of collagen deposition. The amide-I and amide-II absorbances, which come from the skin’s protein composition, were measured for this purpose. The degree of collagen deposition was estimated using this unique technique that compared the absorbance of the treatment group with the control group. The results from three analyses of each sample were averaged.

##### Histology

Animals were killed by cervical dislocation when needed, and the skin-containing wound was surgically removed, cleaned with normal saline, and stored at −20 °C until further usage. Histological testing was conducted on the newly repaired skin tissue that covered the incision. The stored skin samples were thawed at room temperature for 3 h, then fixed in a 10% aqueous solution of formalin for three days at ambient temperatures. The skin samples were then prepped by cutting, washing with regular saline, and dehydrating in ethanol. The samples of desiccated skin were cleaned with xylene before being fixed in paraffin wax. Using a microtome (HM-340E, Microm Inc., Boise, ID, USA), 5 µm thick sections were created. They were then processed separately by Masson’s trichrome and H&E (hematoxylin and eosin) stains. The slides were observed, and relevant portions were photographed employing an inverted microscope equipped with a camera (HDCCE—X5N).

### 2.3. Statistical Analysis

At a minimum, three data replicates were used to calculate the mean and standard deviation. The significance level was established at *p* < 0.05, and the analysis of variance (ANOVA) followed by post hoc analysis or a Student’s *t*-test was employed for analysis.

## 3. Results and Discussion

### 3.1. Moisture Adsorption

**Figure 1:**
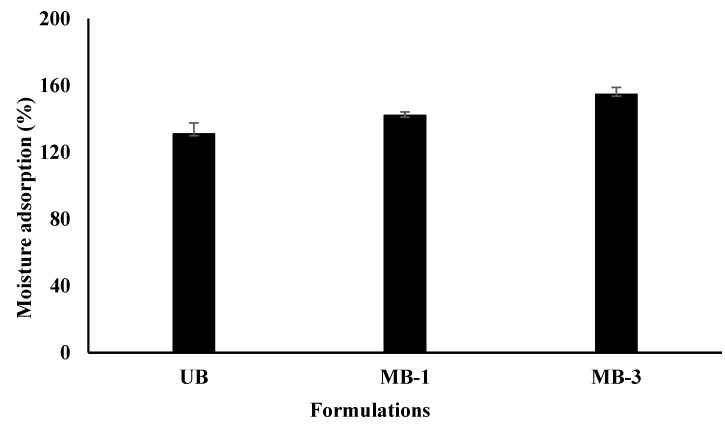
Percentage moisture adsorption of blended film formulations.

An ideal wound-healing platform requires a hydrophilic extracellular matrix that can remove wound exudate and keep the wound bed moist for rapid regeneration [79,92,93]. To check a material’s capacity to hold sufficient moisture in the wound bed, various methods, such as water contact angle and water retention, can be used [9]. The moisture adsorption test results indicated that the percentage moisture adsorption of blended films ranged from 131 ± 6.6% to 154.6 ± 4.23%. The moisture adsorption ability was found to increase significantly with an increase in microwave treatment time, as shown in Figure 1. The increase in water adsorption ability of polymer blend film following microwave treatment can be attributed to free OH functional groups shifting to the surface area, thereby promoting the water attacking ability of the film [94].

Furthermore, microwave treatment is envisaged to increase the crosslinking density between the polymers and other film ingredients such as glycerol, which has been reported to immobilize between polymer chains, resulting in increased water absorbency and enhanced water moisture adsorption ability of the film [88]. Microwaves are polarphilic electromagnetic waves that interact with a polymer’s polar regions in a volumetric manner [95]. Following microwave treatment, the polar functional groups of sodium alginate and sodium carboxymethyl cellulose (i.e., OH, amide-I, and amide-II) may interact via hydrogen bonding [96], forming a compact structure. Moreover, the strong interlinks between the polymer chains cause the polymer fibers to arrange themselves in a uniform/even manner during drying, resulting in voids forming between polymer chains and leading to distinct pore sizes throughout the polymer matrix [97]. Reduced pore size renders maximum moisture absorption at the wound surface [98]. It may decrease the penetration of bacteria into the wound bed, thus preventing complications in the wound [99].

### 3.2. Water Vapor Transmission Rate (WVTR) and Water Vapor Permeability (WVP)

**Figure 2:**
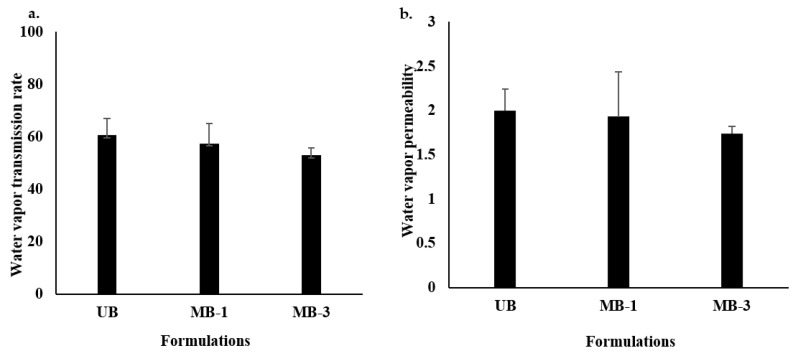
(**a**) Water vapor transmission rate; (**b**) water vapor permeability across various film formulations.

The *WVTR* of a wound healing platform determines its efficiency in reducing the transepidermal water loss and facilitating the easy exchange of oxygen and carbon dioxide between the wound bed and external environment [100], which is inversely proportional to a wound dressing’s ability to retain moisture, implying that a dressing having a low *WVTR* will be capable of retaining more moisture at the wound surface since dry wounds take longer time to heal [101]. The results of *WVTR* are shown in Figure 2a, where though the difference between all formulations was insignificant (Student’s *t*-test, *p* > 0.05), microwave-treated blends tend to have lower *WVTR* compared with the UB formulation, where MB-3 was found to have significantly lower *WVTR* compared with UB (Table 2). Similarly, the *WVP* was highest for UB compared with MB-1 and MB-3 (Figure 2b). More water prevention ability by microwave-treated films could be attributed to specifically engineered pore size due to the arrangement of polymer layers/fibers in a specific geometric manner, probably allowing gaseous passage (O_2_ and CO_2_) through but preventing water molecules passage due to large molecular size [89], which was reported earlier to be due to the initiation of strong interactions between the film moieties following microwave treatment, probably in the form of strong hydrogen bonds [94]. Additionally, microwave irradiation of polymers is also reported to enhance the inter-polymer cross-linking, resulting in enhanced intermolecular forces that arrange the polymers into a better orientation [57]. Furthermore, this phenomenon is envisaged to prevent bacterial infiltration, thereby reducing the chances of secondary bacterial infection by opportunistic bacteria [102].

### 3.3. Erosion and Water Uptake

**Figure 3:**
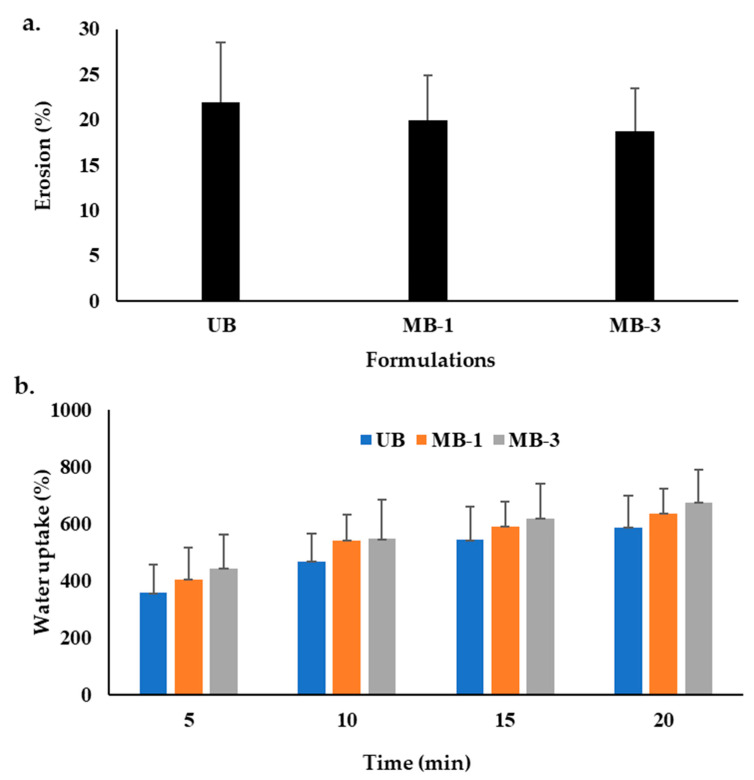
(**a**) Percentage erosion, and (**b**) Water uptake ability of various film formulations.

The water uptake capacity regulates the film formulation’s swelling, degradability, functionality, and stability [103], which are governed by pH, type, and ions at the wound bed. Delayed erosion and high water uptake are deemed favorable from the perspective of skin regeneration, where delayed erosion translates into better patient compliance and a long duration of action. In contrast, water uptake ability reflects the ability of the film formulation to remove exudates from the wound bed [73]. The percentage erosion results of all formulations are shown in Figure 3a, while water uptake is shown in Figure 3b. The results indicated that the UB formulation degraded up to 21.87 ± 6.62%, while microwave treatment reduced the percent erosion ability up to 18.69 ± 4.74%, though the difference was statistically insignificant (Student’s *t*-test, *p* > 0.05). In the case of UB, which was composed of untreated sodium alginate and Na-CMC blend, it is more likely to expose more hydrophilic surface groups due to loosening structure formation as a result of relaxed polymer chains, allowing the easy penetration of erosion media into the matrix, resulting in hastened solubility and hence quick erosion [104]. Conversely, following microwave treatment, a densely cross-linked structure might have been formed, resulting in egg box formation between sodium alginate and Na-CMC polar functional groups [105], thereby offering higher resistance to water penetration into a polymer matrix and delaying matrix damage [89].

The water uptake results indicated that an increase in microwave irradiation time resulted in a higher water uptake capacity with time compared with the untreated blend. However, the difference was insignificant (ANOVA, *p* > 0.05). It is believed that due to the affinity of microwaves towards polar functional groups of the polymers and/or other formulation ingredients, hydrophilic as well as hydrophobic interactions might have resulted in surface shifting of OH/NH, amide, and ester functional groups, enabling more attraction of water molecules, translating into higher water uptake [89], which is envisaged to effectively remove wound exudate and enable faster skin regeneration.

### 3.4. Morphology

**Figure 4:**
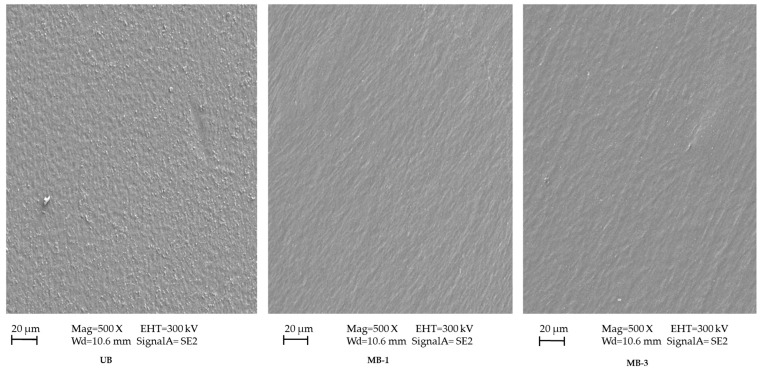
SEM of different blend film formulations.

The surface morphology pictographs of untreated and microwave-treated blend films are shown in Figure 4. The results indicated that the untreated (UB) had a rough/granular appearance, which could be attributed to removing some of the formulation ingredients from the matrix and accumulating on the surface of the mixture as the two polymers were not wholly homogenous and there was minimal separation of the ingredients due to the loose structure of the polymer film. In contrast, the microwave treatment resulted in a more homogenous surface appearance of the film following drying, advocating proper mixing of all formulation ingredients with no phase separation at the interface owing to the microwave’s ability to affect the polymer arrangement [106]. The role of the microwave as an efficient crosslinking agent is augmented by Sun et al. (2018), who described that without microwave treatment, the corn di-starch phosphate/corn straw cellulose film had a rough appearance with a loose structure when viewed cross-sectionally [57]. After microwave/ultrasonic treatment, the surface of the film became homogenous and smooth, with a dense and compact arrangement of polymer chains having no phase separation at the interface. All this happened due to efficient crosslinking between the polymers due to microwave/ultrasonic treatment. Wang et al. (2014) also advocated the role of the microwave as an excellent physical crosslinking method to create the smooth and homogenous appearance of blend films [107].

### 3.5. Tensile Strength

The mechanical strength of a polymeric film reflects its ability to withstand friction and stress during handling and/or application at the wound site [108]. The tensile strength results of all formulations are shown in Table 3. The results indicated that the untreated blend tends to have significantly lower tensile strength than microwave-treated blends (Student’s *t*-test, *p* < 0.05). The tensile strength tends to increase with microwave treatment time; significantly higher (Student’s *t*-test, *p* < 0.05) tensile strength was observed when the blend was subjected to 3 min of microwave treatment. Higher tensile strength is believed to appear due to higher cross-linking density, which shall be optimized as higher cross-linking density translates into a reduction in percent elongation, which may make the film non-elastic [36]. Microwaves affect the physical attributes of polymers such as particle shape, size, distribution, packing style, and diameter, ultimately influencing the substance’s mechanical properties [109]. In a study, microwave-treated soy protein isolate/titanium dioxide film exposed to 500-watt microwave power for 15 min showed maximum tensile strength due to the microwave, which reducing particle size and increased surface area. Increasing the surface area provides an improved opportunity for the particles to interact. Thus, a more stable film is created, which leads to better tensile strength [107]. Sun et al. (2018) showed the same results of improved mechanical strength of corn di-starch phosphate/corn straw cellulose film due to irradiating the film solution with microwaves [57]. They concluded that the increased tensile strength is due to the improved integration of the polymer blend due to enhanced intermolecular force, which amends the molecular structure of the polymer network.

### 3.6. Thermal Analysis

**Figure 5:**
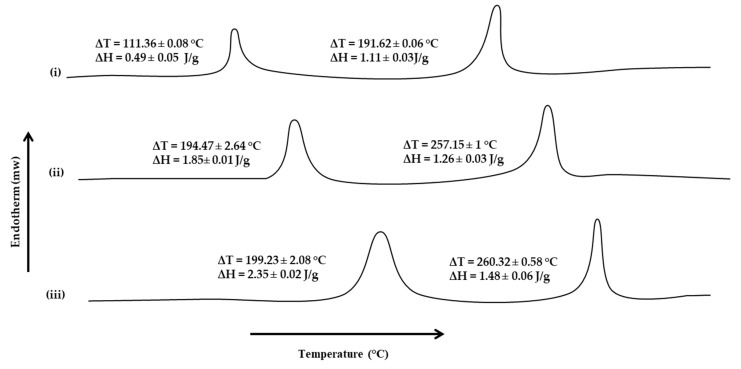
DSC thermograms of (**i**) UB, (**ii**) MB-1, and (**iii**) MB-3.

Thermal analysis, such as DSC, depicts the films’ behavior as a function of temperature and interprets the degradation process, thermal transition, and thermal stability of the films [11]. The DSC thermograms of the blended polymeric films were obtained to describe the thermal properties of films and estimate the effect of microwave treatment on the thermal properties of polymers. The effect of microwaves on the cross-linking ability between sodium alginate and Na-CMC was evaluated by subjecting all formulations to thermal analysis. The DSC thermograms of all formulations are shown in Figure 5. The results indicated that microwave treatment significantly increased the corresponding melting transition temperatures and enthalpies of sodium alginate and Na-CMC moieties. In the case of UB, two transitions were observed, i.e., at 111.36 ± 0.08 °C and 191.62 ± 0.06 °C, with corresponding ∆H values of 0.49 ± 0.05 J/g and 1.11 ± 0.03 J/g, respectively, where the former was attributed to sodium alginate and the latter to Na-CMC moieties. In the absence of microwave treatment, polymer chains become flexible due to surfactant/plasticizer incorporation, due to which molecules move easily, and so less heat is required to reach the glass transition temperature [88]. With the introduction of microwave treatment, the melting transition, as well as corresponding enthalpies, tend to increase. A significant (Student’s *t*-test, *p* < 0.05) rise in the melting transition temperature as well as corresponding enthalpies was observed for MB-3, where the sodium alginate moiety showed ∆T value of 199.23 ± 2.08 °C and ∆H value of 2.35 ± 0.02 J/g. In comparison, for Na-CMC, the ∆T value of 260.32 ± 0.58 °C and ∆H value of 1.48 ± 0.06 J/g were observed. A significant rise in melting transition, as well as the energy required to induce transition, during the thermal analysis of the microwave-treated blend film depicted that microwave treatment enabled the formation of additional interactive forces between both polymer moieties, i.e., electrostatic and/or hydrogen bonding, through both polymer polar functional groups [11,110], thereby requiring higher temperature and energy to induce transition. 

### 3.7. Vibrational Spectroscopic Analysis

**Figure 6:**
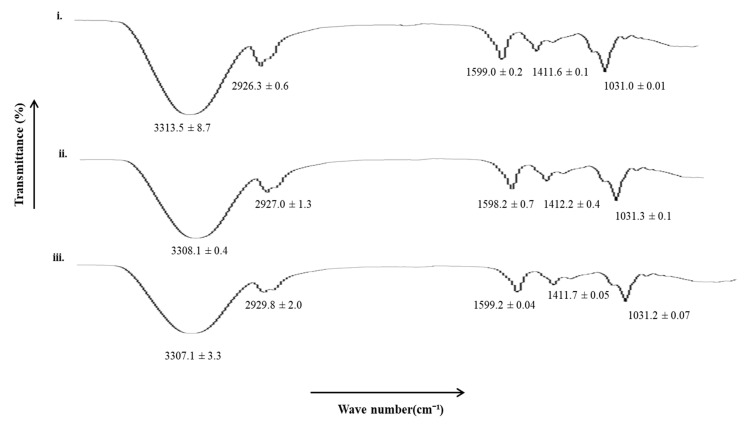
FTIR spectra of (**i**) UB, (**ii**) MB-1, and (**iii**) MB-3.

All the film formulations were subjected to vibrational spectroscopic analysis using an ATR-FTIR to elucidate the extent of hydrophilic and hydrophobic interactions between the polymers and/or polymers and excipients following microwave treatment. The results are shown in Figure 6. The UB film showed characteristic hydrophilic (OH/NH, C=O) and hydrophobic bands (asymmetric CH), which tend to show significant shifts when subjected to microwave treatment. In the case of MB-3, a significant decrease in hydrophilic moieties (OH/NH, 3306–3315 cm^−1^) and a significant increase in hydrophobic moieties (asymmetric CH, 2925–2930 cm^−1^) were observed, depicting rigidification of hydrophilic domains of the film and fluidization/elasticity of hydrophobic domains occurred when films were treated with a microwave for 3 min. The rigidification of hydrophilic moieties of the polymeric blend film could be attributed to the formation of additional linkages between the polar functional groups of both polymers and/or polymers and excipients, which is envisaged to translate into a delay in erosion ability. In contrast, fluidization of hydrophobic domains is predicted to increase the elasticity of the matrix, translating into higher mechanical strength when the polymer blend was treated with microwaves for 3 min.

### 3.8. Wound Morphology

**Figure 7:**
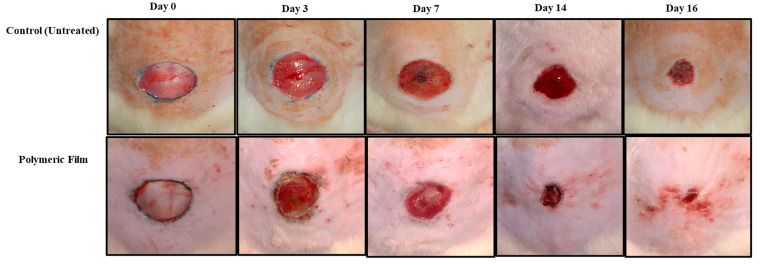
Photographs of skin wound morphology with and without film treatment in diabetic rats.

**Figure 8:**
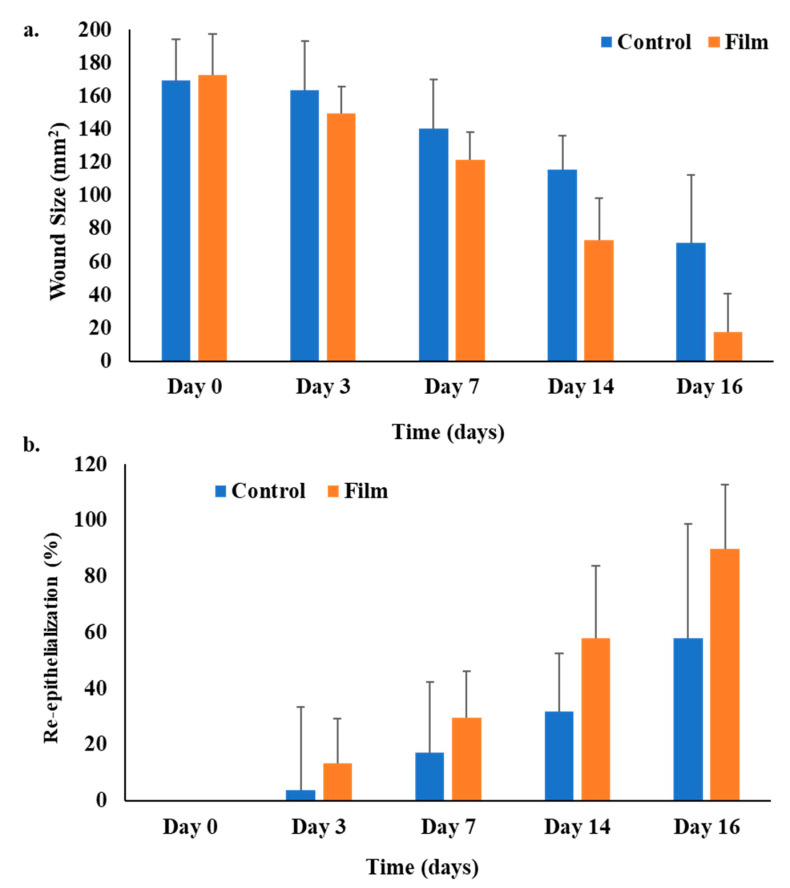
(**a**) Wound sizes (mm^2^) and (**b**) percentage re-epithelialization in the diabetic rats.

The in vivo wound healing ability of the microwave-treated polymer film group and the untreated control group was tested in the diabetic rat model. The pictographs of wound morphology are shown in Figure 7, and wound size and percent re-epithelialization results are in Figure 8a,b. The in vivo evaluation of wounds indicated that the untreated control group did not heal entirely for up to 16 days. Only 58% re-epithelialization was observed, while the polymeric film group showed a prominently high percentage of re-epithelialization (89.7%). The polymeric film group significantly hastened the skin tissue regeneration in diabetic animals in comparison to the untreated/control group with a significantly reduced wound size (ANOVA, *p* < 0.05, Figure 8a), where almost near-complete wound healing (90%) was achieved within 16 days of the experiment with nearly no scar. The absence of scarring can be attributed to the *WVP* of the film scaffold. As stated earlier, the sodium alginate-sodium CMC film scaffold showed an optimal range of *WVP*; thus, wound therapy in a wet environment positively influenced re-epithelization; hence, it encourages healing with no scar development [111]. The polymeric film group resulted in 89.7% re-epithelialization on day 16, which was significantly higher (ANOVA, *p* < 0.05) as compared with that found for the untreated control group (58%). Among both test groups, the polymeric film proved significantly efficient in healing diabetic wounds.

Re-epithelialization, a characteristic hallmark of cutaneous wound contraction, is governed by the restoration of skin tissues to form a granulation barricade on the open wound [112]. The wound healing in terms of contraction and percent re-epithelialization presented promising results in the polymeric film group, which encouraged the process of wound contraction in less time than the control/untreated group. The polymeric film group also supported wound healing better, with 89.7% re-epithelialization, due to the inherent wound-healing nature of films constituting polymers (alginate and CMC) [113]. In wound healing, increased division and migration of epithelial cells along with keratinocytes occur from the periphery of the wound towards the wound site, both of which depend upon the interaction of keratinocytes with the extracellular matrix at the wound surface [114].

### 3.9. Physicochemical Characterization Tests Results of Skin Samples

#### 3.9.1. Thermal Analysis

**Figure 9:**
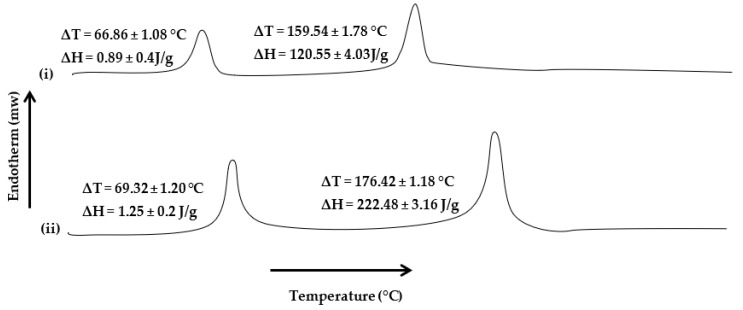
DSC thermograms of rat’s skin without (**i**, **ii**) with polymeric film treatment.

The thermal analysis results of skin samples harvested on the 14th day of post-wounding from both animal groups are presented in Figure 9. Thermal analysis was performed to investigate the extent of collagen deposition during the healing process, which is envisaged to either increase or decrease in the transition melting temperature and enthalpy of the proteinous domains in the skin samples. The results indicated that in both group samples, the melting transition, as well as corresponding enthalpies, did not differ significantly (Student’s *t*-test, *p* > 0.05), where the untreated skin samples showed ∆T = 66.86 ± 1.08 °C, with corresponding ∆H = 0.89 ± 0.4 J/g, while in the film-treated group, it appeared to be 69.32 ± 1.20 °C, with corresponding enthalpy ∆H = 1.25 ± 0.2 J/g. In contrast, in the case of proteinous domains, a significant increase in the melting transition temperatures and enthalpies was observed in samples harvested from the film-treated group compared with the untreated group. The transition temperature significantly increased from 159.54 ± 1.78 °C to 176.42 ± 1.18 °C (Student’s *t*-test, *p* < 0.05), with a significant rise in enthalpy (Student’s *t*-test, *p* < 0.05, ∆H = 120.55 ± 4.03 J/g to 222.48 ± 3.16 J/g). The rise in melting transition and enthalpies of skin protein domains in the film-treated group advocates forming a compact and cross-linked protein structure at the wound site. Sodium alginate and Na-CMC are already reported to possess wound-healing properties [11,62,86]. Microwave treatment might have enabled controlled pore size formation in the polymer matrix, which facilitated skin regeneration by accelerating the process of collagen deposition, which is envisaged to promote rapid wound closure.

#### 3.9.2. Tensile Strength

Uniform and greater extents of collagen deposition are expected to significantly increase the mechanical strength of the newly regenerated skin tissue at the wound site. Therefore, the skin samples harvested on the 14th day of wounding were subjected to tensile strength analysis, and the results are shown in Table 4. The results indicated that the film-treated group showed a significant increase in the tensile strength and percent elongation break compared with the untreated animal group (Student’s *t*-test, *p* < 0.05). The increased tensile strength indicates a compact and dense arrangement of collagen protein in skin structure due to the rigidification of the dermal layer’s hydrophilic moieties (NH/OH, C=O, C-N), depicting the development of a more dense skin structure [97,115].

#### 3.9.3. Vibrational Spectroscopy

**Figure 10:**
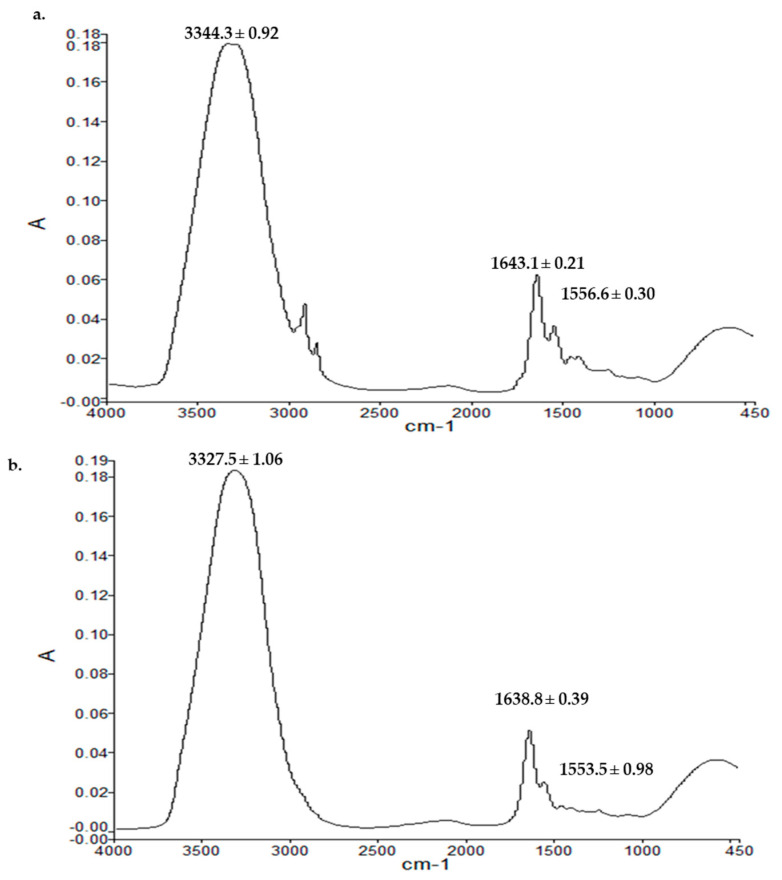
ATR-FTIR spectra of the dermis of (**a**) untreated skin and (**b**) film-treated skin.

The ATR-FTIR spectra of the dermal layer of skin samples from the untreated control group and polymeric film-treated animal groups are demonstrated in Figure 10. The ATR-FTIR analysis was performed to cement the results obtained with the thermal and tensile strength analyses. For this purpose, the wavenumbers with the corresponding absorbance ratios of the OH/NH, amide-I, and amide-II bands were investigated. The amide-I region in the skin is reported to be associated with collagen protein (1650 cm^−1^, C=O stretching in O=C–N–H), while amide-II bands (1550 cm^−1^, N–H bending in O=C–N–H) have been reported to arise from peptide linkages of collagen [116,117]. As shown in Figure 10, the OH/NH absorbance band underwent significant rigidification in the film group (Student’s *t*-test, *p* < 0.05, 3344.3 cm^−1^ to 3327.5 cm^−1^) compared with the untreated control group (Figure 10a). Similarly, the amide-I experienced significant rigidification (Student’s *t*-test, *p* < 0.05), where the absorbance band underwent a significant shift to a lower wavenumber region of 1638.8 from 1643.1 cm^−1^, with similar changes observed with the amide-II (Student’s *t*-test, *p* < 0.05, 1556.6 to 1553.5 cm^−1^). To further strengthen this claim, a ratio of absorbance values of corresponding bands of untreated to film-treated skin was calculated, which was found to be significantly higher for film-treated groups (Student’s *t*-test, *p* < 0.05, amide-I to amide-II = 1.98 ± 0.02), showing more rigidity of hydrophilic moieties in the dermis compared with the untreated group (1.20 ± 0.04). The high wavenumbers and absorbance ratios indicated the rigidity of hydrophilic OH/NH parts of the dermal layer, describing the development of a denser skin structure [97] and a greater extent of protein deposition at the wound site [118].

#### 3.9.4. Skin Histology

**Figure 11:**
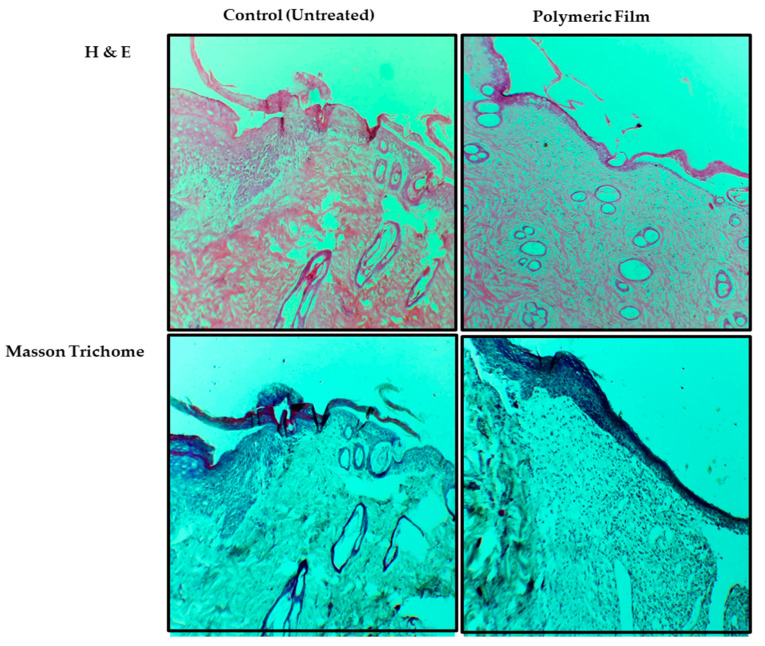
Photomicrographs showing the histological analysis of wound healing at day 14 after H&E and Masson trichrome staining at ×10 magnification.

Histological analysis results of the H&E and Masson trichrome staining are shown in Figure 11. The H&E was performed to visually analyze the inflammatory phase of both animal group samples, while Masson trichrome was employed to investigate the extent and pattern of collagen deposition. As shown in Figure 11, the untreated H&E staining revealed significant inflammation yet on day 14, which was evident from signs of ulceration, edema with loose dermal layer crust, low epithelization, and an abundance of mononuclear cell infiltration compared with film-treated skin samples, with a lesser extent and nonuniform collagen deposition. In contrast, the film-treated samples showed a diverse level of granulation tissue formation, epithelium migration over the dermis, dermal remodeling, lesser edema, ulceration, and a fair quantity of granulation where signs of healed skin structure with fine-shaped were observed with close to normal epidermis, adnexa restoration, and extensive and uniform collagen fiber deposition. The animals treated with the film group presented almost complete wound re-epithelialization, together with well-formed and distinguished epithelium and substantially augmented accumulation of connective tissue and collagen within the dermis.

Similarly, as evident in the results of Masson trichrome, film-treated samples showed a higher amount of collagen accumulation along with proper orientation at the wound site [118]. Wound contraction and healing occur due to inflammatory markers principally activating the keratinocytes and fibroblast cells to hasten the development of the collagen and extracellular matrix, forming the skin tissue’s stroma [119].

## 4. Conclusions

The present study investigated the effectiveness of microwaves in physically cross-linking two natural polymer blends to improve the resulting film’s physicochemical properties from the perspective of wound healing application. The results demonstrated that treating sodium alginate and Na-CMC blend with a fixed frequency of 2450 MHz microwave at a fixed power for 3 min improved the physicochemical properties of individual polymers, thus customizing polymer properties in the form of increased moisture adsorption, low water vapor permeability and water vapor transmission rate, delayed erosion, high water uptake, increased mechanical strength, and homogeneous and uniform surface morphology. These properties were achieved due to tailored pore size and enhanced interaction and compatibility between polymers, facilitating the exchange of oxygen and carbon dioxide between the wound bed and the external environment, preventing enhanced water loss from the wound, which is envisaged to promote healing. Moreover, in the in vivo study, the microwave-modified (MB-3) blend film hastened the skin tissue regeneration, with rapid wound closure, increased collagen deposition, and higher percent re-epithelization compared with the untreated group. Combining sodium alginate and sodium CMC with microwave treatment in film formulation may open new horizons in skin tissue regeneration applications in diabetic wound treatment.

## Figures and Tables

**Table 1:** Composition of modified blended sodium alginate and sodium CMC film formulations.

Formulations	Microwave Treatment Time (min)	Sodium Alginate (*w*/*w*) g	Na-CMC (*w*/*w*) g	Tween 80 (*w*/*w*) G	PEG-400 (*w*/*w*) G	Glycerol (*w*/*w*) g	Water (*w*/*w*) G
Untreated blend (UB)	---	2	2	0.1	0.05	2	93.85
MB-1	1	2	2	0.1	0.05	2	93.85
MB-3	3	2	2	0.1	0.05	2	93.85

**Table 2:** *WVTR* and *WVP* along with standard deviation of blend films.

Sodium Alginate and NaCMC Blend Films			
Formulation	*WVTR* (g/m^2^/h)	*WVP* (g mm/h/m^2^)	The Thickness of the Film (mm)
UB	60.7 ± 6.2	2.00 ± 0.24	0.78 ± 0.01
MB-1	57.5 ± 7.7	1.93 ± 0.51	0.80 ± 0.01
MB-3	53.0 ± 2.8	1.74 ± 0.08	5.12 ± 0.03

**Table 3:** Tensile strength values of different blend film formulations.

Formulation	Tensile Strength (MPa)	Elongation at Break (%)	Elastic Modulus (MPa)
UB	40.54 ± 1.02	65.34 ± 2.53	57.12 ± 10.76
MB-1	48.06 ± 1.30	69.13 ± 2.87	65.88 ± 9.87
MB-3	56.84 ± 1.19	77.54 ± 1.59	79.26 ± 7.68

**Table 4:** Tensile strength of skin in various treatment groups.

Tested Groups	Tensile Strength (MPa)	Elongation at Break (%)	Elastic Modulus (MPa)
Untreated	7.43 ± 1.13	11.09 ± 0.32	1.49 ± 1.71
Polymeric film	12.4 ± 1.02	16.71 ± 0.21	3.84 ± 1.32

## Data Availability

All data about this project has been presented in this manuscript.
